# Evaluation of focal cartilage lesions of the knee using MRI T2 mapping and delayed Gadolinium Enhanced MRI of Cartilage (dGEMRIC)

**DOI:** 10.1186/s12891-016-0941-y

**Published:** 2016-02-11

**Authors:** Asbjørn Årøen, Helga Brøgger, Jan Harald Røtterud, Einar Andreas Sivertsen, Lars Engebretsen, May Arna Risberg

**Affiliations:** Department of Orthopedic Surgery, Akershus University Hospital, 1478 Lørenskog, Norway; Oslo Sports Trauma Research Center, The Norwegian School of Sport Sciences, Pb 4014 Ullevål Stadion, Oslo, Norway; Institute of Clinical Medicine, Campus Ahus, University of Oslo, 1478 Lørenskog, Norway; Department of Radiology, Oslo University Hospital Ullevål, Kirkeveien 166, 0450 Oslo, Norway; Department of Orthopedic Surgery, Diakonhjemmet Hospital, Pb 23, Vindern, 0319 Oslo, Norway; Department of Orthopedic Surgery, Oslo University Hospital Ullevål, Kirkeveien 166, 0450 Oslo, Norway; Norwegian Research Center for Active Rehabilitation, Department of Orthopedic Surgery, Oslo University Hospital Ullevål, Kirkeveien 166, 0450 Oslo, Norway

**Keywords:** Knee, Cartilage lesion, MRI, dGEMRIC, T2-mapping

## Abstract

**Background:**

Assessment of degenerative changes of the cartilage is important in knee cartilage repair surgery. Magnetic Resonance Imaging (MRI) T2 mapping and delayed Gadolinium Enhanced MRI of Cartilage (dGEMRIC) are able to detect early degenerative changes. The hypothesis of the study was that cartilage surrounding a focal cartilage lesion in the knee does not possess degenerative changes.

**Methods:**

Twenty-eight consecutive patients included in a randomized controlled trial on cartilage repair were evaluated using MRI T2 mapping and dGEMRIC before cartilage treatment was initiated. Inclusion was based on disabling knee problems (Lysholm score of ≤ 75) due to an arthroscopically verified focal femoral condyle cartilage lesion. Furthermore, no major malalignments or knee ligament injuries were accepted. Mean patient age was 33 ± 9.6 years, and the mean duration of knee symptoms was 49 ± 60 months. The MRI T2 mapping and the dGEMRIC measurements were performed at three standardized regions of interest (ROIs) at the medial and lateral femoral condyle, avoiding the cartilage lesion

**Results:**

The MRI T2 mapping of the cartilage did not demonstrate significant differences between condyles with or without cartilage lesions. The dGEMRIC results did not show significantly lower values of the affected condyle compared with the opposite condyle and the contra-lateral knee in any of the ROIs. The intraclass correlation coefficient (ICC) of the dGEMRIC readings was 0.882.

**Conclusion:**

The MRI T2 mapping and the dGEMRIC confirmed the arthroscopic findings that normal articular cartilage surrounded the cartilage lesion, reflecting normal variation in articular cartilage quality.

**Study identifier:**

NCT00885729, registered April 17 2009.

**Electronic supplementary material:**

The online version of this article (doi:10.1186/s12891-016-0941-y) contains supplementary material, which is available to authorized users.

## Background

Full-thickness articular cartilage lesions in the knee represent a major health problem, which is reflected by the fact that it is diagnosed in 10 % of all knees subjected to knee arthroscopy [1] and often occurs in younger age groups [1, 2]. Surgical treatment options do exist; however, they have not been proven to provide superior results compared with the natural history of the condition. Even though the majority of both surgically treated and untreated patients experience an improvement in knee function over time, knee function are seldom restored to normal [3, 4].

To assess changes in cartilage before surgery, Magnetic Resonance Imaging (MRI) is the established method of choice in addition to arthroscopic evaluation. Attempts have been made to standardize the biomechanical evaluation of cartilage using different indention probes, but the resulting methods are not commonly used [5]. However, early degenerative changes in the surrounding cartilage without substance loss are difficult to assess; for example, chondrocyte harvesting from the edge of the lesion revealed inferior results to the more standard biopsy [6]. MRI T2 mapping and the delayed Gadolinium Enhanced MRI of Cartilage (dGEMRIC) have been used in trials to assess cartilage quality before joint preserving surgery [7] and in the follow-up of repair procedures [8]. Though MRI T2 mapping is capable of assessing collagen organization [9], the dGEMRIC technique can indirectly reflect the proteoglycan concentration [10, 11].

The aim of the current study was to evaluate whether the cartilage surrounding a focal articular cartilage lesion was assessed as normal by an arthroscopic probing using MRI T2 mapping and dGEMRIC. The ipsilateral non-injured condyle and the contra-lateral knee were analyzed for comparison and served as controls. The current study hypothesized that the cartilage surrounding a focal cartilage lesion is assessed as normal through an arthroscopic evaluation. This means that no differences can be detected in comparison to the rest of the articular cartilage in the ipsilateral knee joint. Additionally, a comparison to the articular cartilage in the contra-lateral knee joint was made and values recorded by the dGEMRIC readings of the articular cartilage were compared with symptom scores of knee pain as the most prominent symptom of degenerative changes of articular cartilage.

## Methods

### Patient population

The patient cohort included in the current study represents patients with a cartilage procedure indicated in clinical practice according to the approval of the Norwegian Regional Committee for Medical and Health Research Ethics (REC) South East. Patients included in a randomized controlled trial (RCT) (www.clinicaltrials.gov: NCT00885729) on cartilage repair were subjected to evaluation by MRI T2 mapping and a dGEMRIC to test the study hypothesis prior to treatment. 72 (22 %) of 332 eligible patients were included in the original RCT. Of the included 72 patients, 28 (39 %) consecutive patients underwent MRI T2 mapping and dGEMRIC. The inclusion criteria for the RCT were disabling knee problems due to a focal femoral condyle cartilage lesion (International Cartilage Repair Society [ICRS] grade 3 or 4) (http://www.cartilage.org), a Lysholm score of ≤ 75 and an age between 18 and 50 years.

The exclusion criteria were major malalignments (more than 6° varus or valgus) and knee ligament injuries. Prior to inclusion, all patients were assessed with a knee arthroscopy procedure to grade and assess the cartilage lesion area and the surrounding cartilage and to exclude other reasons for the knee symptoms.

### Standing radiographics

Standing radiographics at inclusion were taken at the start of the study and categorized according to the guidelines for Kellgren Lawrence with grades ranging from 0 to 4 [12]. All of the patients were scored by a radiology specialist (H.B.).

### MRI protocol

The MRI imaging was performed using a 1.5 T Siemens Avanto MRI machine (Siemens Healthcare GmbH, Erlangen, Germany). The patients were examined using a standard knee MRI protocol [13] that was developed and used at Oslo University Hospital and included T2 mapping and a dGEMRIC [14]. The patients were scanned with all of the different sequences mentioned in Additional file 1, except for the last sequence of the T1 map [15]. Then, they were injected with a 2 mmol/kg bodyweight dose of Gadopentetate dimedlumine, Gd-DPA (Magnevist, Berlex Laboratories, Wayne, NJ, US) [16]. The contrast agent was injected in an antecubital vein. After the injection, the patients walked for 15 min and then rested for approximately 75 min before the last scan was performed [17].

### MRI evaluation

**Fig. 1:**
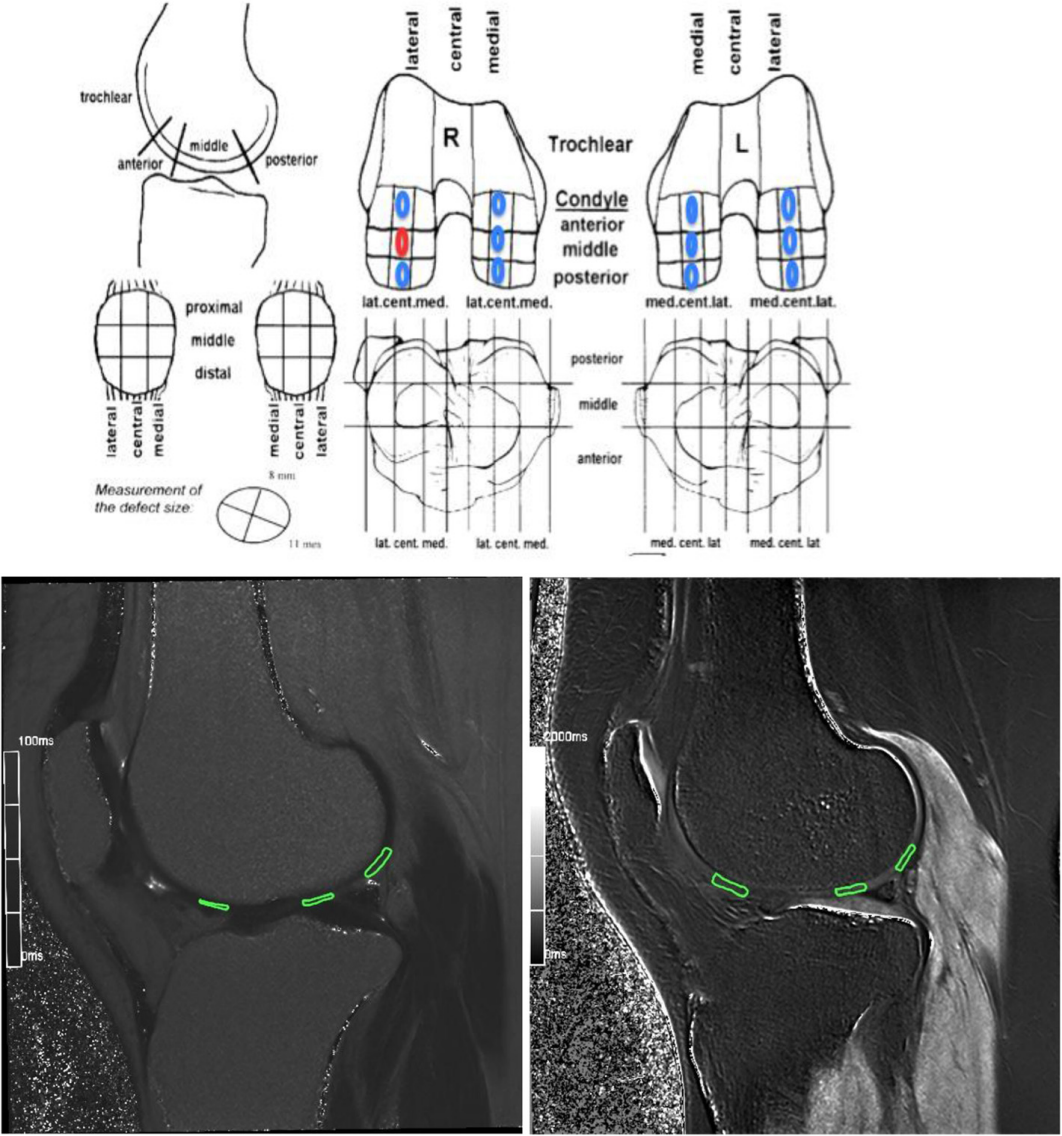
Modified standard drawing published by the International Cartilage Repair Society (ICRS) for mapping a cartilage lesion. The red area describes cartilage injury, and the corresponding blue areas describe the reference point of measurements of the injured versus non-injured knee and condyle. Underneath is a corresponding illustration of the ROIs in T1 (left) and T2 (right) of the lateral condyle of the same knee

All evaluations were performed by a radiologist (H.B.) who was blinded from the x-ray readings and all other clinical information. The MRI scans were examined using an OSIRIX DICOM viewer on a Mac AirBook PRO. The quantitative T1-post-contrast relaxation time measurements were performed on central sagittal sections in both condyles. The MRI T2 mapping and the dGEMRIC measurements were conducted in standardized areas outside the focal cartilage lesion at the three regions of interest (ROIs) at each condyle in both knees (for the T2 mapping, only the affected knee was analyzed). The ROIs were defined and drawn as follows: the anterior ROI stretched from the end of the anterior horn of the menisci to a line drawn cranially from the anterior border of the tibia plateau; the central ROI included the posterior part of the area between the anterior and posterior menisci; and the posterior ROI spanned from the end of the posterior horn of the menisci to a line drawn cranially from the posterior border of the tibia plateau. These regions are illustrated in Fig. 1. The ROIs included the entire cartilage thickness from the subchondral bone to the surface. To compare the injured condyle with the corresponding contralateral condyle, the average of three standardized measurements was used as an assessment of general cartilage health, similar to a previous publication in hip cartilage assessment [7]. Any intraobserver variance of the MRI dGEMRIC readings was evaluated by analyses that were conducted three weeks apart.

### Knee symptoms

The Knee Injury and Osteoarthritis Outcome Score (KOOS) is considered a valid, reliable and responsive self-administered questionnaire for patients with several types of knee injury and knee OA [18]. It has been validated for ACL and cartilage injuries and for other knee injuries [19–21]. It consists of five subscales: pain, symptoms, activities of daily living (ADL), function in sports and recreation (Sport/Rec), and knee-related quality of life (QoL). The KOOS has been frequently used to evaluate knee function in studies on cartilage repair [21, 22]. Thus, the KOOS was obtained in the current study to allow for comparisons with other cohorts of patients with cartilage lesions of the knee. In addition, the KOOS was used to investigate the relation between patient-reported knee pain and the dGEMRIC results of the affected condyle.

### Ethics

This study was approved by the Norwegian Regional Committee for Medical and Health Research Ethics (REC) South East (110-07038a 1.2007.67), and all patients signed an informed consent prior to inclusion.

### Statistics

A one-way ANOVA was used for the statistical evaluation of the primary parameter of the study. According to previous studies that used the same methodology to assess degenerative changes of the hip articular cartilage, a difference of interest of >100 ms in the dGEMRIC values of the injured condyle compared with the contralateral normal condyle was considered indicative of a loss of the cartilage organization ultrastructure. A loss of proteoglycan, as reflected by a reduction of 100 ms in the dGEMRIC values relative to healthy cartilage, has previously been reported to be indicative of osteoarthritis [7]. In the current project, the dGEMRIC analyses used corresponding points on each condyle. The ROIs were manually chosen and standardized at three different points in each knee condyle, as illustrated in Fig. 1, and the values were reported as the mean and the 95 % CI. Unpaired Student’s t-tests were used for the MRI T2 mapping results because these results were not calculated for the contralateral knee. A linear regression was used to analyze the relationship between the dGEMRIC readings of the injured condyles and both the duration of symptoms and the subscale of KOOS pain. A paired *t*-test was used to compare the mean of the injured condyle with the contralateral control condyle. The intraclass correlation coefficient (ICC) was calculated to assess the intraobserver reliability of the dGEMRIC assessments.

## Results

The 28 patients included had a mean Lysholm score of 49 (range, 29–75). The mean size of the cartilage lesions was 2.7 cm^2^ (range, 1–6 cm^2^), and the lesions were located on the medial femoral condyle in 19 of the patients and on the lateral femoral condyle in 9 patients. Unilateral knee problems were noted in 25 of the 28 knees, and for the three patients who reported bilateral problems, these problems were related to a previous meniscus injury and the feeling of catching at times in the contralateral knee. The mean age was 33 ± 9.6 years, and the mean duration of knee symptoms was 49 months (range, 2–240 months). 18 of the patients reported acute onset of symptoms, with a mean duration of symptoms of 29 months (range, 2–120 months). The mean time period between the arthroscopic evaluation and the MRI was 251 days (range, 22–2006 days). The patient demographics of the current study group are reported in Table 1.

**Table 1:** Patient characteristics

Age, years	33 ± 9.8
Male/Female	17/9
BMI	26 ± 4.3
Lysholm score (mean ± SD)	49 ± 22
Tegner score (median (range))	1.0 (0–6)
Lesion size, cm^2^	2.7 ± 1.2
Previously failed microfracture treatment	8
Duration of symptoms, months	49 ± 60
Number of patients out of work due to knee problems	15
KOOS Pain	55 ± 16
KOOS Symptoms	58 ± 17
KOOS Activities of Daily Living	71 ± 16
KOOS Sports/Recreation	21 ± 16
KOOS Quality of Life	27 ± 18

### MRI T2 mapping

**Fig. 2:**
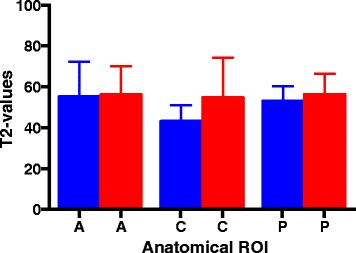
Mean values (ms) of MRI T2 mapping of the medial femoral condyles. The blue bars represent the unaffected medial condyles, and the red bars represent the affected medial condyles. ROI, region of interest; A, anterior; C, central; P, posterior

**Fig. 3:**
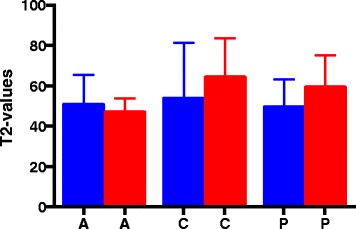
Mean values of the T2 mapping of the lateral femoral condyles. The blue bars represent the unaffected lateral condyles, and the red bars represent the affected lateral condyles. ROI, region of interest; A, anterior; C, central; P, posterior

The results from the MRI T2 mapping are illustrated in Figs. 2 and 3. The MRI T2 mapping did not demonstrate significant differences between the mean values of the articular cartilage on the affected medial condyles and the unaffected medial condyles at the anterior reference point (injured mean 55.2 ms ± 5.7 versus mean 53.5 ms ±2 .7) (*p* = 0.76), central reference point (injured mean 43.1 ms ± 2.7 versus mean 55.8 ms ± 5.2) (*p* = 0.08), or posterior reference point (injured mean 53.1 ms ± 2.4 versus mean 56.5 ms ± 2.0) (*p* = 0.34).

### dGEMRIC

None of the dGEMRIC measurements showed significant differences between the affected and unaffected femoral condyles (Tables 2, 3 and 4). The dGEMRIC results for the injured medial condyle versus the medial condyle of the non-injured knee are reported in Table 2. The results for the lateral condyle showed similar results (Table 3). The average dGEMRIC assessment (mean of the three reference points) for the injured and corresponding control condyles did not reveal a significant difference (mean difference 35.5 ms, 95 % CI, −1.1-72.2, *p*-value = 0.057) (Table 4). The intraobserver reliability revealed an ICC of 0.882 for two separate dGEMRIC value readings by the same radiologist, which is in line with previously published data for this method [23].

**Table 2:** Mean dGEMRIC values (ms) for both medial femoral condyles in patients with a cartilage lesion located at the medial femoral condyle in the corresponding point of measurements; A = anterior region, C = central region and P = posterior region of the condyle

ROI	Control MC	Non Injured MC	Injured MC	*p*-value
	Mean ± SD	Mean ± SD	Mean ± SD	
A	399 ± 85	386 ± 47	398 ± 81	*p* =0.82
C	478 ± 101	453 ± 86	437 ± 89	*p* = 0.29
P	467 ± 101	450 ± 88	452 ± 88	*p* =0.73

*ROI* region of interest, *SD* standard deviation; *p*-value, level of significance – one way ANOVA with Bonferroni corrections (*p*-value less than 0.01 for significance). The control MC represents the medial femoral condyle in the non-affected knee, the non-injured MC represents the values in the medial femoral condyle in the knee in which the lateral condyle had a cartilage lesion, and the injured medial MC is the condyle where the cartilage lesion is located

**Table 3:** Mean dGEMRIC values (ms) for the lateral femoral condyle

ROI	Control LC	Non Injured LC	Injured LC	*p*-value
	Mean ± SD	Mean ± SD	Mean ± SD	
A	411 ± 98	383 ± 54	375 ± 68	*p* = 0.40
C	499 ± 109	491 ± 131	439 ± 96	*p* = 0.34
P	486 ± 94	477 ± 87	455 ± 125	*p* = 0.20

The control LC represents the lateral femoral condyle in the non-affected knee, the non-injured LC represents the values in the lateral femoral condyle in the knee in which the lateral condyle had a cartilage lesion, and the injured lateral LC is the condyle where the cartilage lesion is located

*ROI* region of interest, *SD* standard deviation; *p*-value, level of significance – one way ANOVA with Bonferroni corrections (*p*-value less than 0,01 for significance)

**Table 4:** Mean dGEMRIC values (ms) for the lateral femoral condyle and the medial femoral condyle outlining the injured and control condyles

ROI	Control dGEMRIC	Injured dGEMRIC	Difference: control-injured (95 % CI)
LC-A	410 ± 99	374 ± 67	21.7, 95 % CI (−58.5-101.8)
LC-C	501 ± 103	438 ± 96	53.6, 95 % CI (−66.7-174.0)
LC-P	473 ± 99	455 ± 125	9.4, 95 % CI (−97.9-116.7)
MC-A	404 ± 87	394 ± 78	32.8, 95 % CI (−8.7-73.7)
MC-C	483 ± 103	437 ± 98	56.9, 95 % CI (−21.1-134.8)
MC-P	462 ± 97	440 ± 87	28.4, 95 % CI (−34.0-90.7)

### dGEMRIC, KOOS and duration of symptoms

**Fig. 4:**
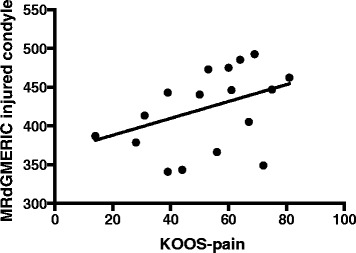
The dGEMRIC readings (mean of three different measurements in the condyle) in the injured condyle in relation to the KOOS pain score values reported by the patients at the dGEMRIC examination. The best fit line shows a significant deviation, *p*-value = 0.039

**Fig. 5:**
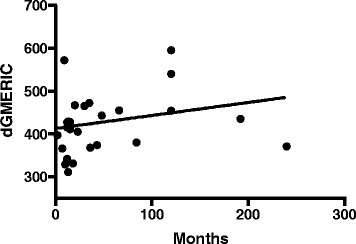
The dGEMRIC readings (mean of three different measurements in the condyle) in the injured condyle in relation to the duration of symptoms. The best fit line does not show a significant deviation, *p*-value = 0.08

A significant correlation (*p*-value = 0.04) was found between the slope of the line relative to the value of KOOS pain and the dGEMRIC readings (average of the three ROIs), as illustrated in Fig. 4. According to the regression analyses (*p*-value = 0.08), the duration of knee symptoms did not reveal a significant association with decreased dGEMRIC readings of the injured condyle, as illustrated in Fig. 5.

### Radiographics

A Kellgren Lawrence grade 0 condition was present in 13 patients, grade 1 was present in 10 patients, and grade 2 was present in 4 patients.

## Discussion

The major finding of the current study was that the articular cartilage surrounding a focal cartilage lesion of the knee that was graded as arthroscopically normal was verified using a dGEMRIC and MRI T2 mapping to show no significant degeneration compared with the opposite knee, even with an average duration of 4 years of symptoms. This finding is in line with a long term follow-up study on autologous chondrocyte transplantation (ACI), in which non-significant changes were found between the repair tissue and the surrounding cartilage [8]. This is also in line with a study of knees with ACL injuries that used dGEMRIC analyses and found no differences between the injured and non-injured knees after non-operative handling of the ACL injury for 20 years. However, in that study, the patients revealed high functional outcome scores, in contrast to the patients in the current study [24]. In studies focusing on the hip joint, other authors have found that the changes diagnosed with dGEMRIC eventually pass a critical point after which surgery cannot reverse the progression towards the need for a hip replacement. In the current study, all of the mean readings were greater than 382 ms, and based on studies of the hip joint, a threshold of 350 ms has been suggested to indicate a later need for hip replacement [7]. The same threshold of 350 ms has also been found to indicate progression to radiological osteoarthritis in the knee [25]. The current study observation is in accordance with a previous meta-analysis indicating that the need for knee replacement after cartilage repair involving ACI was less than 1 % [26]. However, it is possible that some knees with cartilage lesions can pass a threshold in proteoglycan depletion of the articular cartilage that cannot be reversed by cartilage surgery. To outline this possibility, a recurrent and longer follow-up of patient cohorts with cartilage lesions of the knee using specific cartilage MRI protocols is needed. However, it is interesting to note that the subscale of KOOS pain was significantly associated with the dGEMRIC readings (Fig. 4); this finding supports those of other studies in which the dGEMRIC readings were found to correlate with both knee extensor and flexor strength measurements [27]. This association is further supported by a recent publication [28] showing that the unloading of the joint resulted in inferior dGEMRIC values in the unloaded joint; the pain experienced by the patients with knee loading due to their cartilage defects may be the reason for this finding in our study.

Based on the findings of the current study, cartilage that is arthroscopically graded as normal will also be evaluated as normal in a dGEMRIC analysis.

The aim of the current study was to reveal whether a difference greater than the level of interest (100 ms) in the dGEMRIC readings could be found between the injured cartilage and the normal condyle in both injured and contralateral knees. The observed difference in the current study was far less and was not statistically significant. This study reported the preoperative dGEMRIC values of articular cartilage in the injured knee; to the best of our knowledge, these results represent new data, although the same technology has been used in the long-term follow-up of surgically treated patients to obtain favorable results after surgery in relation to cartilage quality [8].

The limitations of the current study are the relatively low number of patients, the wide range in the duration of symptoms, and the 3 patients with bilateral knee problems. However, these limitations are very similar to those found in several clinical trials on cartilage repair [29]. All three patients with bilateral knee problems reported a prior treated meniscus injury, which is not uncommon in this age group. Additionally, the dGEMRIC is sensitive in detecting ultrastructural changes, which are otherwise impossible to assess without several biopsies in different areas of the knee joint [30, 31]. The T2 and dGEMRIC values are subjected to some intraobserver variance, as reported in the current study; this should be kept in mind when evaluating cartilage repair techniques and utilizing modern MRI evaluation as an endpoint. In a more methodologically aimed study, the reproducibility of the dGEMRIC measurements has generally been found to be good [23]. The material presented in the current study represents a patient group in which cartilage repair is indicated in clinical practice, as stated in the methods section. The findings reported indicate that a focal articular cartilage lesion induces limited degenerative changes in the surrounding cartilage, and not to a degree that cannot be recovered in a successful cartilage repair surgery [32]. Thus, the present study suggests that the cartilage surrounding a cartilage lesion is, in most cases, not the limiting factor of success after cartilage repair surgery.

## Conclusion

Evaluated by MRI T2-mapping and dGEMRIC, the presence of a focal cartilage lesion did not result in significant changes in the surrounding cartilage. Based on the findings in the current study, there seems to be a good correlation between the arthroscopic grading and the dGEMRIC evaluation of the cartilage surrounding a cartilage injury.

## Additional file

Additional file 1:
**Scanprotocol dGEMRIC.** (DOC 28 kb)
