# Supplementary material for: Evaluation of focal cartilage lesions of the knee using MRI T2 mapping and delayed Gadolinium Enhanced MRI of Cartilage (dGEMRIC)
Source: BMC Musculoskelet Disord. 2016 Feb 11;17:73. doi: 10.1186/s12891-016-0941-y (PMC4751750; doi:10.1186/s12891-016-0941-y)
Supplement: Additional file 1: — Scanprotocol dGEMRIC. (DOC 28 kb) [file 12891_2016_941_MOESM1_ESM.doc]

**Additional file 1:**

**Scanprotocol dGEMRIC.**

**Cor PD TSE FS**

TR 3190 ms

TE 29 ms Restore pulse

FOV 140mm.

Matrix: 346 x 384

Voxel size: 0.4 x 0.4 x 3.0 mm.

Slice thickness: 3mm.

**Sag PD TSE FS**

TR 3660 ms

TE 51 ms  Blade

Matrix: 384 x 384

Voxel sixe: 0.4 x 0.4 x 3.0 mm.

Slice thickness: 3 mm.

**Axial PD TSE FS**

TR 4800 ms

TE 28 ms Restore pulse

FOV 140 mm.

Matrix: 240 x 320

Voxel size: 0.5 x 0.4 x 3.0 mm.

Slice thickness: 3 mm.

**Sag PD TSE**

TR 3650 ms

TE 24 ms

FOV 140 mm

Matrix: 346 x 384

Voxel size: 0.4 x 0.4 x 3.0 mm.

Slice thickness: 3 mm.

**CorT1 SE**

TR  512 ms

TE 11 ms

FOV 160 mm.

Matrix: 307 x 384

Voxel size: 0.5 x 0.4 x 3.0 mm.

Slice thickness: 3 mm.

**High Resolution:**

**Cor  PD TSE**

TR 3540 ms

TE 33 ms Restore pulse

 FOV 100 mm.

Matrix: 320 x 320

Voxel size: 0.3 x 0.3 x 2.5 mm.

Slice thickness: 2.5 mm.

**High Resolution:**

**Sag PD TSE**

TR 3690 ms

TE 29 ms Restore pulse

 FOV 100 mm.

 Matrix: 266 x 320

Voxel size: 0.4 x 0.3 x 2.0 mm.

Slice thickness: 2 mm.

**T2 Map**

**Sag  T2 SE (spin ekko**)

TR 1200

TE 1: 12.7 ms

TE 2: 25.4 ms

TE 3: 38.1 ms

TE 4: 50.8 ms

TE 5: 63.5 ms

TE 6: 76.2 ms

FOV 125 mm

Matrix: 320 x 320

Voxel size: 0.4 x 0.4 x 4.0 mm.

Slice thickness: 4 mm.

**T1 Map 3D (fl3d_vibe)**

**Sag  T1FL (Flash = Fast Low Angle Shot)**

TR 15 ms

TE 4.76 ms

Flip angle 1: 5 deg

Flip angle 2: 26 deg

FOV 140 mm

Matrix: 320 x 320 mm.

Voxel size: 0.4 x 0.4 x 4.0 mm.

Slice thickness : 4 mm.

T1 estimate: 800 ms
